# Trust Beliefs in Significant Others, Interpersonal Stress, and Internalizing Psychopathology of Adolescents with Psychiatric Disorders

**DOI:** 10.1007/s10578-021-01255-x

**Published:** 2021-10-02

**Authors:** Ken J. Rotenberg, Carla Sharp, Amanda Venta

**Affiliations:** 1 School of Psychology, Keele University, Keele, UK; 2 Clinical Psychology, University of Houston, Houston, USA; 3 Sam Houston State University, Houston, USA

**Keywords:** Adolescents, Psychiatric disorders, Trust beliefs, Interpersonal stress, Internalizing psychopathology

## Abstract

This study examined relations between trust beliefs in significant others (TBSO), interpersonal stress, and internalizing psychopathology for adolescents with psychiatric disorders. Two hundred and thirty-four adolescents from an acute inpatient unit (154 females, *M*_age_ = 14.72 years, *SD* = 1.39 years) completed standardized scales/interviews that assessed TBSO (mother, father, teacher, peers and TBSO as a total score), interpersonal stress, and internalizing psychopathology. It was found that adolescents’ TBSO and trust beliefs in each significant other were negatively associated with interpersonal stress and internalizing psychopathology. The findings confirmed that the relation between the adolescents’ interpersonal stress and internalizing psychopathology was moderated by TBSO, trust beliefs in mother, and trust beliefs in peers. The findings supported the conclusion that holding elevated TBSO (particularly trust beliefs in mother and peers) by adolescents with psychiatric disorders promotes their psychosocial adjustment including their resilience to the effects of interpersonal stress on internalizing psychopathology.

## Introduction

Interpersonal trust is regarded as essential to the psychosocial adjustment of adolescents [1]. Interpersonal trust by adolescents from the community is associated with their mental and physical health. It buffers them from the effects of social stress on mental health and physical health. Interpersonal trust increases the adolescents’ resilience to the effects of stress [2–4]. The studies, however, often assess broad measures of interpersonal trust and mental health. There is a scarcity of research on interpersonal trust for adolescents with psychiatric disorders. Interpersonal trust may be particularly important for them because of their heightened interpersonal stress, internalizing psychopathology and poor family relationships [5–7]. The current study was guided by Attachment Theory [8] and the Basis, Domain and Target theoretical framework [9]. It was designed to fill in a gap in knowledge by examining whether interpersonal trust beliefs in significant others (TBSO, mother, father, and peers) by adolescents with psychiatric disorders: (a) are negatively associated with interpersonal stress and internalizing maladjustment and (b) moderates (and buffers) the relation between their interpersonal stress and internalizing maladjustment.

### Interpersonal Stress and Internalizing Psychopathology During Adolescence

Adolescents are prone to interpersonal stress which encompasses conflicts with friends, financial problems, and academic difficulties [6, 10]. Adolescents are also prone to internalizing psychopathology such as anxiety and depression [10–12]. Several studies show that girls demonstrate greater interpersonal stress [13–16] and greater internalizing psychopathology than boys [17–21]. Interpersonal stress is a probable cause of internalizing psychopathology during adolescence. Adolescents’ interpersonal stress is concurrently and prospectively associated with internalizing symptoms [17–21]. Adolescents with psychiatric disorders demonstrate elevated interpersonal stress and internalizing psychopathology which contributes to their disorder [22, 23].

### Attachment Theory and Interpersonal Trust

The research on interpersonal trust has been frequently conceptualized and examined within the context of Attachment Theory [8]. According to this theory, when infants establish a secure attachment, they form an Internal Working Model (IWM) of parent–child relationship that predisposes the child to social competence and psychosocial adjustment across development. The IWM includes a sense of trust in others and positive thoughts regarding the intentions of other people’s behaviors [24, 25] and generalizes beyond caregivers to others, including teachers [26] and peers [27]. De Winter et al. [28] found that early adolescents who held high expectations for supportive attachment figures (as a measure of trust in them) demonstrated a positive bias in interpreting maternal behavior. Meta-analyses have shown that early security of attachment is linked to greater social competence and fewer internalizing psychopathology [29, 30].

### Trust Beliefs in Others and Psychosocial Adjustment

Interpersonal trust beliefs in others by adolescents from the community are associated with lower-levels of mental problems and help them cope with mental problems [2–4]. Adolescents with psychosis and those with externalizing psychopathy display limitations in their capacity to develop trust during game interactions [31, 32]. TBSO, specifically their mother, father or peers has been found to play a positive role in psychosocial adjustment by adolescents from the community. TBSO by those adolescents is associated with higher levels of mentalization, higher levels of communication with parents, lower levels of aggression, higher levels of psychological well-being, lower levels of internalized maladjustment and lower levels of alienation [33–36]. Venta et al. [37] found that emotional trust beliefs in mothers by adolescents with psychiatric disorders’ moderated, and thus served as a buffer of, the relation between depression and suicide attempts. There is a scarcity of research on the relation between TBSO, interpersonal stress and internalized psychopathology by adolescents with psychiatric disorders.

The current research was guided by the Basis, Domain, and Target framework of interpersonal trust (BDT) [9, 38]. According to this framework, TBSO comprises individuals’ expectations that those persons show reliability (e.g., fulfil promises), honesty (e.g., tell the truth), and emotional trustworthiness (e.g., refrain from causing emotional harm). The BDT interpersonal trust framework provides an insight into how TBSO promote psychosocial adjustment. Adolescents who hold high TBSO would expect for example that significant others would: (a) fulfill their promises to assist them in resolving emotional problems, (b) honestly convey information about conflict-laden events to them, and (c) be acceptant and supportive of the disclosure of their negative emotions. Such expectations would increase the capacity for adolescents to cope with high interpersonal stress.

Guided by the preceding BDT formulations and Attachment Theory [8, 29, 30] we expected that trust beliefs in the TBSO and each significant other (mother, father, teacher, and peers) by adolescents with psychiatric disorders would be negatively associated with their interpersonal stress and internalizing psychopathology. It was expected that the TBSO and trust beliefs in each significant other would moderate, and thus buffer, the relation between interpersonal stress and internalizing psychopathology. The latter would provide support for the principle that those trust beliefs increased the adolescents’ resilience to the effects of interpersonal stress on their internalizing psychopathology.

### Overview of the Current Study and Hypotheses

In the study, adolescents in an acute inpatient psychiatric unit were administered standardized measures/interviews assessing TBSO, interpersonal stress, and internalizing psychopathology.

The following hypotheses were advanced:

1. Adolescents’ TBSO and trust beliefs in each significant other would be negatively correlated with interpersonal stress and internalizing psychopathology. The latter two variables would be correlated. (*Hypothesis 1*: *Trust Belief and Adjustment*).
2. TBSO and trust beliefs in each significant other would moderate and buffer the relation between interpersonal stress and internalizing psychopathology. When adolescents experienced high interpersonal stress, they would demonstrate lower levels of internalizing psychopathology when they held high rather than low trust beliefs across significant others and each significant other (*Hypothesis 2*: *Trust Buffering*).
3. Guided by gender differences in previous research, it was expected that girls would report greater interpersonal stress than boys and demonstrate greater internalizing psychopathology than boys (*Hypothesis 3*: *Gender Differences*).

Analyses were carried out to examine whether there were gender differences and in the expected associations and moderation.

## Method

### Participants

Each of 355 consecutive admissions to the 16-bed adolescent unit of a county psychiatric hospital located in a large diverse metropolitan area in the United States was approached for consent on the day of admission. Of those approached, 36 declined, 64 were discharged prior to completion of the assessments (given the acute nature of most admissions to the unit), 5 began assessments and then revoked consent, and 16 were excluded from the study (see Procedure). Therefore, the original sample was reduced to 234. Approximately 65.8% of the sample (*n* = 154) was female and the average age was 14.72 years (*SD* = 1.386). The sample was ethnically diverse and the ethnic/racial breakdown was as follows: 40.6% Hispanic, 27.4% African-American, 25.2% Caucasian, 4.7% Multiracial, 0.9% Southeast Asian, and 1.3% who identified as “Other.”

The participants had been hospitalized during the period of 2010 to 2013 with the majority hospitalized during 2011 (44%). The adolescents are admitted for to the hospital for depression, anxiety disorder, substance use problems, oppositional defiant disorder, and conduct disorder. Clinical diagnoses of the adolescents were not available but the participants were administered the Beck Depression Inventory, BDI [39] and the Youth Self Report, YSR [40]. The diagnostic categories of the BDT are 0–13 is minimal, 14–19 is mild, 20–28 is moderate, and 29–63 is severe. 28% had moderate depression, 30% had severe depression. 62% exceeded the cut-off point for the YSR Internalizing, and 58% exceeded the cut-off for YSR Externalizing.

### Procedures

This study was approved by the appropriate institutional review boards. The inclusion criteria adopted were English fluency (because of the English scales), voluntary admission to the hospital, age between 12 and 17 years, and capacity to participate in research (i.e., absence of severe psychosis or intellectual/developmental disabilities). The stress interviews were carried out by doctoral students under supervision of, and with periodic visits, by an investigator. The interviewers were blind to the diagnostic status of the adolescent.

### Measures

#### Trust Beliefs in Significant Others

The 24-item Children’s Generalized Trust belief scale (CGTB; Rotenberg et al. [41]) assessed the extent to which individuals believe that significant others (i.e., mother, father, teacher, and peers) demonstrate reliability, emotional trustworthiness, and honesty. The participants imagined they were protagonists who were of the same-gender in vignettes. The following are examples of the vignettes (items) presented to girls (with the target and basis identified in brackets): “Lorraine’s father said that he would take her to the cinema on Saturday. How likely is it that Lorraine’s father will take her to the cinema? (*Father-Reliability*)”.“Tina tells her Mother that she held hands with a boy at school, but asks her Mother not to tell anyone. How likely is it that Tina’s Mother will not tell others about it? (*Mother-Emotional*).” The participants judged each vignettes on a 5-point Likert scale ranging from 1 very unlikely to 5 very likely. The complete CGTS is reprinted in Rotenberg [38].

The English, Italian and Chinese CGTS demonstrate construct validity by three-factor/bases by factor analyses and acceptable internal consistency with αs > 0.70 [41–43]. TBSO (mother, father, teacher, and peers) are correlated and are associated with a common construct of TBSO. Shortened forms or subscales of the English CTGS have been used by Gordon et al. [44] to examine children’s secret keeping and by Rotenberg et al. [45] to examine the quality of children’s peer interactions in the playground. The CGTB has been employed to assess trust beliefs of early adolescents [46] and adapted to assess trust beliefs for late adolescents [47]. The TBSO in the current study demonstrated acceptable internal consistency across items α = 0.79. Also, each of the TBSO subscales demonstrated acceptable internal consistency with α = 0.63, 0.68, .54, and .54 for mother, father, teacher, and peers, respectively. The internal consistencies need to be viewed in light of the limited number of items per subscale and the multifactorial nature of the measure. Higher scores denoted greater trust beliefs on the total scale and in each of the four significant others subscales.

#### Interpersonal Stress

This was assessed by the semi-structured UCLA Life Stress Interview [12, 48]. This semi-structured interview was administered by trained research staff and prompts adolescents to describe aspects of their lives (e.g., social life) and queries about sources of stress occurring in that domain. Interpersonal stress comprised the chronic stress participants had with close friends, social (life), romantic relationships, and familial relationships. The stressors from the interview narrative were coded by group discussions composed of three doctoral students and the interviewer. The respondent’s level of chronic stress in each of several domains was assigned a rating of 1 (none) to 5 (severe) according to the rating criteria prepared by Hammen [12]. Because these domains are not rated as items of a composite scale, internal consistency was not calculated. Greater numbers on the scale denoted greater interpersonal stress.

#### Internalizing Psychopathology

This was assessed by the Anxious Depressed and Withdrawn Depressed subscales of the YSR [49] and the BDI-II [39]. The YSR is a self-report questionnaire for use with adolescents between the ages of 11 and 18. The items are scored on a 3-point scale (0 = “not true”, 1 = “somewhat or sometimes true”, or 2 = “very or often true”). These scales were derived from the 112 problem items of the YSR based on principal components analysis [50] and have been found to correspond highly with clinician diagnosis [40]. In the current study, adolescents completed the YSR on a computer and it was electronically scored. Therefore, item-level data is not available for internal consistency analyses.

The BDI-II also was used as an indicator of internalizing psychopathology. This 21-item self-report inventory assesses the severity of depressive symptoms. Each item is rated on a 0–3 scale and, thus, total scores range from 1 to 63. The internal consistency, factor structure, and validity of BDI-II have been previously evaluated [39] and among adolescents, the measure has been used with adequate validity (e.g., Cronbach’s α = 0.92 [51]). In the present study, Cronbach’s α for the BDI-II was 0.93. There were substantive correlations between the three measures (i.e., YSR Anxious Depressed, YSR Withdrawn Depressed, and BDI-II) of internalizing psychopathology with rs ranging from 0.60 to 0.73 (dfs = 232, and ps < 0.001). Internalizing psychopathology was calculated by summing the three measures. The resulting internalizing psychopathology score was subjected to lngamma transformation in order to normalize its distribution. Raw means for this measure are presented in Table 1 as a frame of reference. Higher scores designated greater internalizing psychopathology.

**Table 1:** Correlations between the measures (with *Ms* and *SD*s)

*Measure*	M	SD	TBM	TBF	TBT	TBP	IS	IP
Trust beliefs in significant others								
Total (TBSO)	75.28	12.83	0.55***	0.76***	0.75***	0.72 ***	− 0.25***	− 0.34***
Mother (TBM)	20.02	7.22		0.29***	0.41***	0.30***	− 0.21*	− 0.27**
Father (TBF)	18.93	4.82			0.42***	0.38***	− 0.22**	− 0.19*
Teacher (TBT)	18.07	4.32				0.43***	− 0.18*	− 0.30***
Peers (TBP)	18.66	3.77					− 0.20**	− 0.25**
Interpersonal stress (IS)	11.24	2.98						
Internalizing psychopathology (IP)	39.97	20.60						

*Dfs* = 168 to 178

*p < 0.05, **p < 0.01, and ***p < 0.001

### Data Analysis Strategy

First, MANOVA and ANOVAs with gender as a between factor were carried on the measures to test the Gender differences Hypothesis (Hypothesis 3). Second, correlational analyses were carried out to test for the hypothesized associations between the measures as expected by the Trust Belief and Adjustment Hypothesis (Hypothesis 1). Third, a series of individual Hierarchal Regression Analyses (HRAs) were used to test the Trust Buffering Hypothesis (Hypothesis 2). The predictors in Step 1 of an HRA were interpersonal stress and a given measure of trust beliefs comprising TBSO, *or* trust beliefs in mother *or* trust beliefs in father, *or* trust beliefs in teacher, *or* trust beliefs in peers. Because Attachment Theory proposes that there are *naturally occurring relations* among TBSO, the HRAs were simply carried out on each measure of trust beliefs. The interaction (i.e., multiplication) of the two variables (e.g., TBSO * interpersonal stress *or* trust beliefs in mother * interpersonal stress, or father * interpersonal stress, etc.) were entered as a predictor in Step 2 of the HRA. The dependent measure in each HRA was internalizing psychopathology. The predictors were centered as recommended by Cohen et al. [52]. The data is available for view at the Open Science Framework: https://osf.io/p3b7e/?view_only=3f16c6d1f36a4bf992c8396a62247d7e.

## Results

### Gender Differences

The ANOVA testing on internalizing psychopathology yielded an effect of gender, *F*(1, 168) = 7.04, *p* = 0.009, η^2^ = 0.041. As expected (Hypothesis 3), girls showed greater internalizing psychopathology, *M* = 24.69, *SD* = 1.43, than did boys, *M* = 18.51, *SD* = 1.85. Contrary to Hypothesis 3, the ANOVA on interpersonal stress did not yield gender differences that attained or approached significance. The MANOVA on the TBSO subscales did not yield gender differences that attained or approached significance, *F*(4, 169) = 0.83, *p* = 0.510.

### Correlations Between the Measures

The correlations between the measures (with *Ms* and *SDs*) are shown in Table 1. As expected, there were correlations between: (a) trust beliefs in each of the four significant others; and (b) the TBSO and trust beliefs in each of the four significant others (which contributed to the former measure). As expected, there was a correlation between interpersonal stress and internalizing psychopathology. In support of the Trust Belief and Adjustment Hypothesis (Hypothesis 2), TBSO and trust beliefs in each of the four significant others were negatively correlated with interpersonal stress and internalizing psychopathology. Comparisons of the correlations between genders by z calculations did not yield significance, providing no evidence that the associations between these variables differ by gender.

### Hierarchical Regression Analyses

The HRAs yielded main effects of trust beliefs in mother, β =  − 0.25*, p* = 0.003, trust beliefs in father at a trend level, β =  − 0.17, *p* = 0.058, trust beliefs in teacher, β =  − 0.28, *p* < 0.001, trust beliefs in peers, β =  − 0.23, *p* = 0.009, and TBSO, β =  − 0.33, *p* < 0.001, on internalizing psychopathology with interpersonal stress statistically controlled. These effects complement the observed correlations. The HRAs showed that the main effect of trust beliefs in mother was qualified by trust beliefs in mother and interpersonal stress interaction, β =  − 0.30, *p* = 0.008. Also, the main effect of trust beliefs in peers was qualified by trust beliefs in peers and interpersonal stress interaction that approached statistical significance, β =  − 0.16, *p* = 0.069. The HRA showed that the main effect of TBSO was qualified by a TBSO and interpersonal stress interaction, β =  − 0.17, *p* = 0.04.

**Fig. 1:**
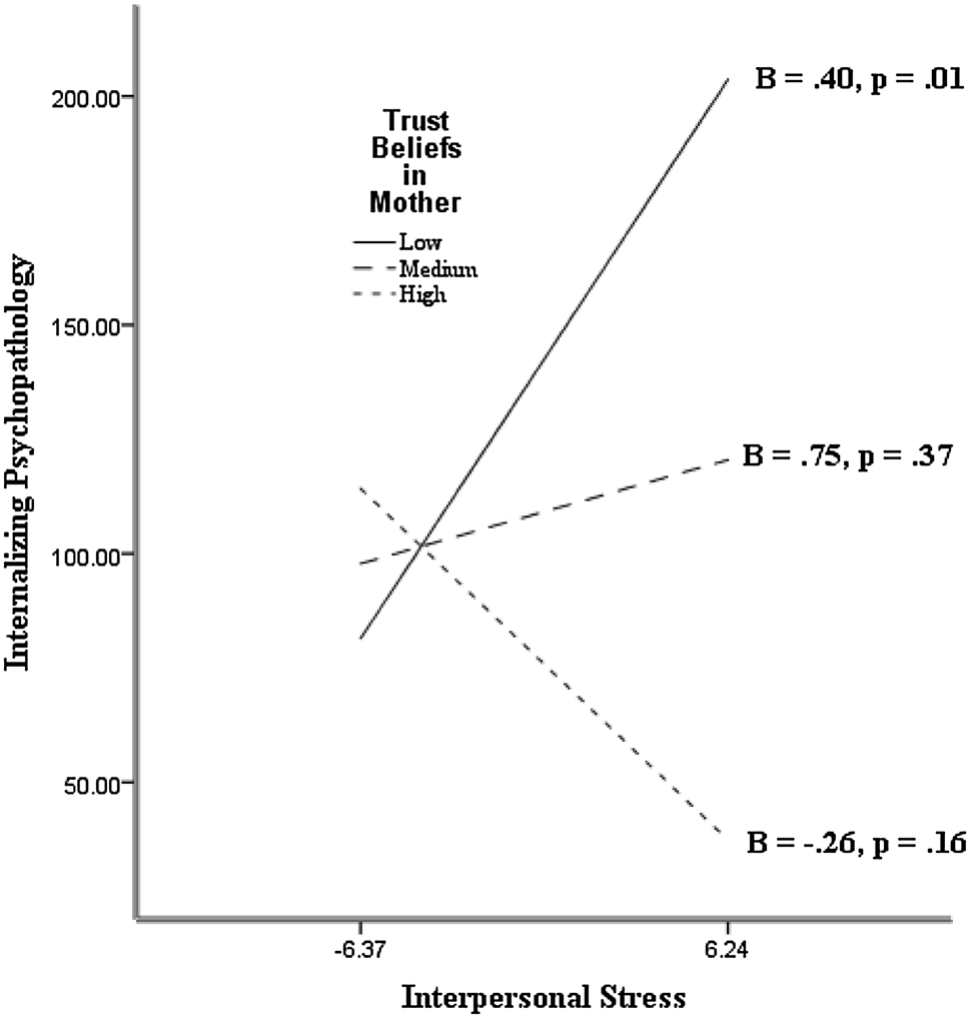
The slopes of the relation between interpersonal stress and internalizing psychopathology as a function of trust beliefs in mother

**Fig. 2:**
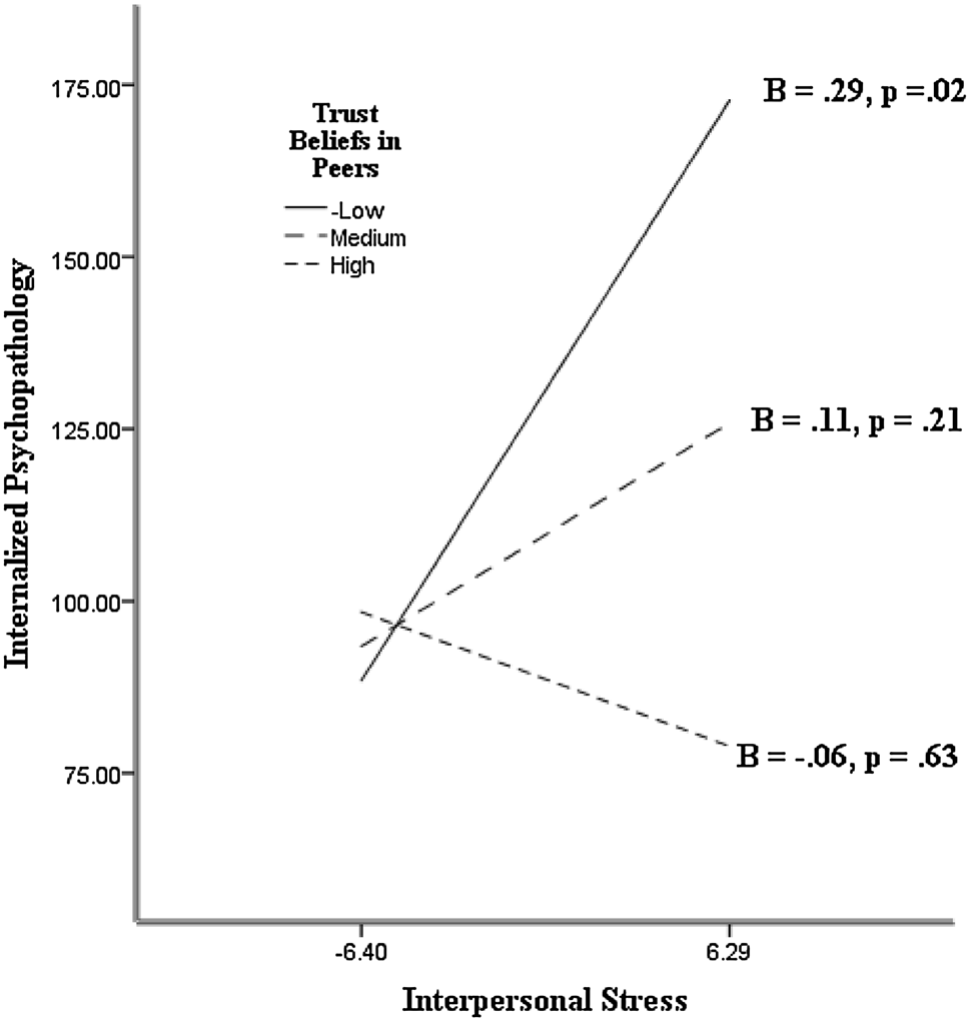
The slopes of the relation between interpersonal stress and internalizing psychopathology as a function of trust beliefs in peers

**Fig. 3:**
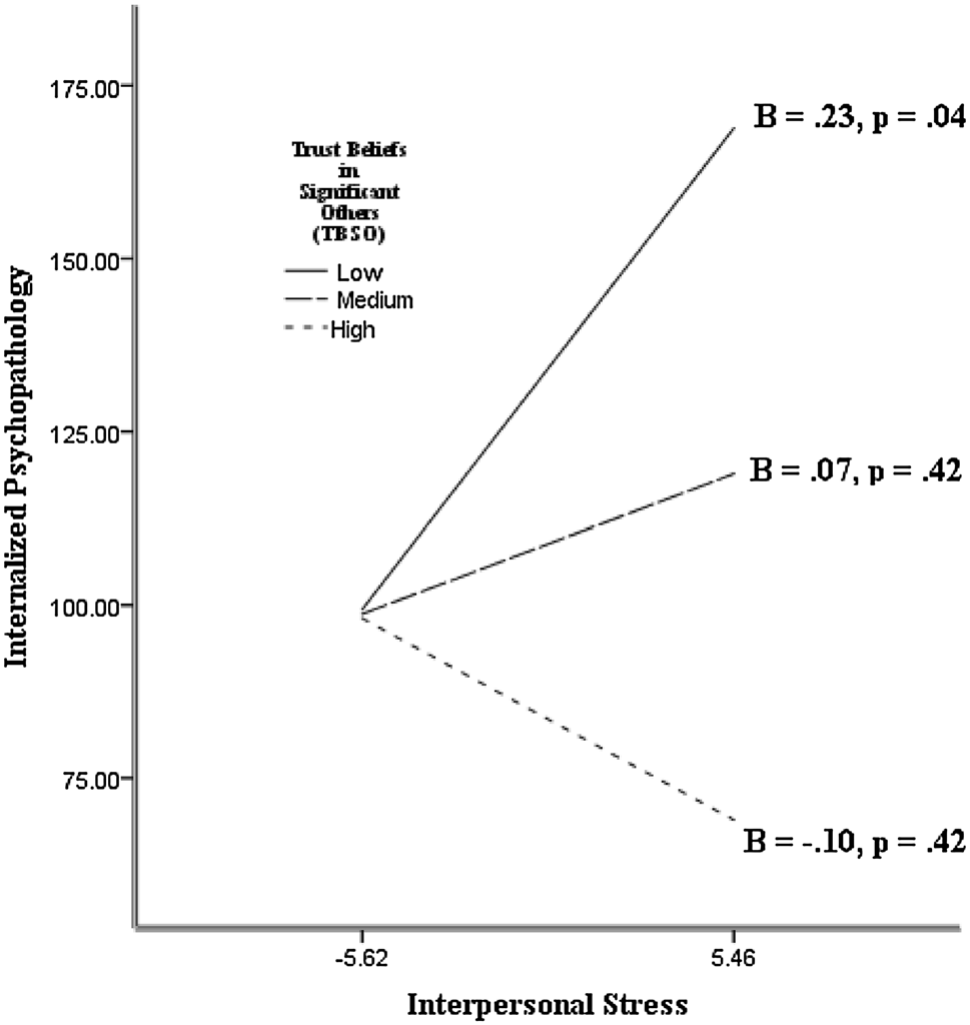
The slopes of the relation between interpersonal stress and internalizing psychopathology as a function of trust beliefs in significant others (TBSO)

The observed interactions were further examined by tests of the slopes of the relation between interpersonal stress and internalizing psychopathology as a function of TBSO, trust beliefs in mother, and trust beliefs in peers (the moderators). The slopes were calculated with the trust beliefs were 1 *SD* above the mean (high), the mean (medium), and 1 *SD* below the mean (low). The slopes of the interaction with trust beliefs in mother, trust beliefs in peers, and TBSO are shown in Figs. 1, 2, and 3, respectively. As expected, there was statistically significant, positive slope between interpersonal stress and internalizing psychopathology when trust beliefs in mother, trust beliefs in peers, and TBSO were low. There was no appreciable relation between interpersonal stress and internalizing psychopathology when TBSO, trust beliefs in mother, and trust beliefs in peers were medium. The slopes were in the positive direction. There was no appreciable relation between interpersonal stress and internalizing psychopathology when trust beliefs in mother, trust beliefs in peers, and TBSO were high. In this case the slopes showed a negative valence. Comparison of the slopes confirms the expectation that, when the participants had high interpersonal stress, they tended to show lower internalizing psychopathology if they held high than low trust beliefs in mother, trust beliefs in peers, and TBSO. The relation between interpersonal stress and internalized psychopathology was absent when trust beliefs in mother and trust beliefs in peers and TBSO were high. The pattern yielded support for the Trust Buffering Hypothesis (Hypothesis 2) across significant others, and specifically for mother and peers as targets of trust. Additional HRAs showed that the observed interactions between trust beliefs and interpersonal stress on internalized psychopathology were not moderated by gender.

## Discussion

The current study examined the relations between interpersonal trust in significant others, interpersonal stress and internalizing psychopathology for adolescents with psychiatric disorders. Gender differences in the measures were examined. In support of the Gender Hypothesis, girls showed greater internalizing psychopathology than boys which replicate previously observed gender differences [13]. Contrary to that hypothesis, the current study did not yield a gender difference in interpersonal stress which has been previously observed [15]. The participants in the study were adolescents from an acute inpatient unit and the lack of gender differences might be due to some distinct aspect of that sample. Their elevated interpersonal stress could have masked potential differences in gender. The lack of observed gender differences in adolescents’ TBSO is consistent with previous research [41].

In support of the Trust Belief and Adjustment Hypothesis, negative associations were found between TBSO and internalized psychopathology for adolescents with psychiatric disorders. These associations were found across TBSO and for each significant other. Also, as expected, interpersonal stress was correlated with internalized psychopathology. This latter finding is consistent with research showing that adolescents’ interpersonal stress is concurrently and prospectively associated with internalized psychopathology [16].

The study yielded support of the Trust Buffering Hypothesis. It was found that trust in significant others moderated and buffered the relation between interpersonal stress and internalized psychopathology. The moderation was found for trust beliefs across significant others, trust beliefs in mother, and trust beliefs in peers at a trend level. When adolescents experienced high interpersonal stress, lower internalized psychopathology was shown if the adolescents held high rather than medium or low trust beliefs. Of particular interest was the finding that adolescents who held high trust beliefs in the significant others did not show the prevailing positive relation between interpersonal stress and internalizing symptoms whereas those who held low trust beliefs did. These findings support the principle that TBSO by adolescents with psychiatric disorders increased their resilience to the effects of interpersonal stress on their internalized psychopathology.

The current findings are consistent with a number of lines of research. The findings complement studies showing that TBSO by adolescents from the community is associated with higher levels of mentalization, higher levels of communication with parents, lower levels of aggression, higher levels of psychological well-being, lower levels of internalized maladjustment and lower levels of alienation [33–36]. The current findings are compatible with research showing that trust beliefs in others buffers the effects of social stress on the mental health and health for adolescents from the community [3, 4]. Finally, the findings complement studies demonstrating that adolescents with some forms of psychopathology (psychosis, externalizing) display limited capacity to development of trust in game interactions [31, 32]. The current study uniquely shows the nature of the relations between interpersonal trust in significant others, interpersonal stress and internalizing psychopathology for adolescents with psychiatric disorders.

The findings yield support for Attachment Theory by showing the adolescents’ trust beliefs in mother primarily served as a moderator and buffer of the relation between interpersonal stress and internalizing psychopathology. The prevailing role of trust beliefs in mothers as a moderator and buffer is consistent with that found by Venta et al. [37] who found that emotional trust beliefs in mothers by adolescents with psychiatric disorders served a similar function in the relation between depression and suicide attempts. The findings also are consistent with the Attachment Theory principle that those trust beliefs generalized to peers as part of the IWM. It was found that the adolescents’ trust beliefs in peers tended to serve a similar buffering function. The absence of evidence for buffering by trust beliefs in father is not consistent, however, with Attachment Theory. There is evidence that relationships with fathers are highly disrupted for adolescents who are inpatients of psychiatric institutions [53] and that could attenuate the buffering effects of trust beliefs in fathers. Although attachment theory has been extended to student–teacher relationships, the relationships between adolescents and their teachers may not be very close and therefore their trust beliefs in teachers did not serve as a buffer.

The BDT interpersonal trust framework [9] provide insights into how TBSO play a positive role in the adolescents’ psychosocial adjustment. When adolescents hold elevated TBSO (notably mother, and peers) they believe that they fulfill promises, convey information honestly, and refrain from causing emotional harm. Such beliefs may help adolescents with psychiatric disorders draw upon social resources (e.g., offers by parents) in order to cope with interpersonal stressors. In support of this principle, it has been found that adolescents’ perceptions of their mothers as trustworthy (e.g., trusted, keeps promises and is honest) predicted the frequency of their talks with mothers about health risks and the adolescents’ risk-taking behavior [35, 54]. It would be worthwhile for researchers and practitioners to include measures of TBSO when attempting to identify protective factors to augment in treatment during that period in development.

There may be a correspondence between the adolescents trust beliefs in parents, and the trustworthiness of parents. For example, lower levels of adolescents’ trust beliefs in mothers could reflect parental patterns of not fulfilling promises, being dishonest, and causing emotional harm towards the adolescent. To our knowledge, this hypothesis has not been directly examined. It has been found that parents of children or adolescents with psychopathology demonstrate lower levels of positive parent–child parenting style, lower-levels of parental involvement, and higher levels of authoritative/punitive disciple [55, 56]. The reliability of a care provider is essential to the development of secure attachment and by implication to trust beliefs in parents [57]. In the future, researchers could examine whether trust beliefs in parents by adolescents with psychiatric disorders function in conjunction with parental trustworthiness to promote psychosocial adjustment. Low trust beliefs and untrustworthiness in particular could serve as an etiology of psychiatric disorders.

The current findings are relevant to the clinical treatment of adolescents with psychiatric disorders. TBSO by adolescents with psychiatric disorders have been regarded as essential to successful clinical treatment [58, 59]. This principle is supported by the finding that adolescents’ trust in their mothers and fathers is predictive of the success of family therapy [60]. The trustworthiness of mothers and fathers may play a complementary role in the final success of family therapy. Specifically, the convergence of the adolescents’ trust beliefs in parents and the trustworthiness of parents could affect recovery from psychiatric disorders. Recovery from psychiatric disorders would be impeded if adolescents’ trust beliefs in significant parents are low and trustworthiness of parents is similarly low.

There are two directions for future research. First, the current study is cross-sectional design and longitudinal research is needed to examine the expected probable causal relations between TBSO, interpersonal stress and internalizing psychopathology in adolescents with psychiatric disorders. Unfortunately, longitudinal research with psychiatric adolescent samples is limited. Other research lends support for the hypothesized causal relations. Longitudinal studies show that TBSO are predictive of symptoms of internalized psychopathology in nonclinical samples of children and adolescents [36]. Longitudinal research with clinical samples is needed.

Second, internalizing psychopathology in the current study was assessed by validated standardized scales. The measures were administered to the adolescents only. It would be worthwhile to utilize other reports (e.g., parents and teachers) and clinical instruments to assess psychopathology in future research.

## Summary

The study yielded relations between TBSO in their social network, interpersonal stress, and internalizing psychopathology for adolescents with psychiatric disorders. The participants were 234 adolescents from an acute inpatient unit (154 females, M_age_ = 14.72 years, SD = 1.39 years). They completed standardized scales/interviews that assessed TBSO (mother, father, teacher, peers and TBSO as a total score), interpersonal stress, and internalizing psychopathology.

It was found that the adolescents’ trust beliefs in each significant other were negatively associated with interpersonal stress and internalizing psychopathology. The study showed that the relation between the adolescents’ interpersonal stress and internalizing psychopathology was moderated, and thus buffered, by their TBSO, primarily mother and peers. The study supported the conclusion that holding elevated TBSO (notably mothers and peers) by adolescents with psychiatric disorders promote their psychosocial adjustment including increased resilience to the effects of interpersonal stress.
